# Peer Reporting: Sampling Design and Unbiased Estimates

**DOI:** 10.3390/e28010116

**Published:** 2026-01-18

**Authors:** Kang Wen, Jianhong Mou, Xin Lu

**Affiliations:** College of Systems Engineering, National University of Defense Technology, Changsha 410073, China; wenkang@nudt.edu.cn (K.W.); moujianhong@nudt.edu.cn (J.M.)

**Keywords:** network sampling, ego-network, statistical inference, activity ratio, complex networks

## Abstract

The Ego-Centric Sampling Method (ECM) leverages individual-level reports about peers to estimate population proportions within social networks, offering strong privacy protection without requiring full network data. However, the conventional ECM estimator is unbiased only under the restrictive assumption of a homogeneous network, where node degrees are uniform and uncorrelated with attributes. To overcome this limitation, we introduce the Activity Ratio Corrected ECM estimator (${\mathrm{ECM}}_{\mathrm{ac}}$), which exploits network reciprocity to recast the population–proportion problem into an equivalent formulation in edge space. This reformulation relies solely on ego–peer data and explicitly corrects for degree–attribute dependencies, yielding unbiased and stable estimates even in highly heterogeneous networks. Simulations and analyses on real-world networks show that ${\mathrm{ECM}}_{\mathrm{ac}}$ reduces estimation error by up to 70% compared with the conventional ECM. Our results establish a theoretically grounded and practically scalable framework for unbiased inference in network-based sampling designs.

## 1. Introduction

Sample surveys are fundamental to quantitative research in social, behavioral, and health sciences, forming the empirical basis for understanding population characteristics and health reasoning [1]. More broadly, recent work in informatics emphasizes that reliable inference often relies on extracting essential structure from incomplete and noisy observations rather than from fully observed data [2,3]. However, the validity of survey data is severely challenged when surveys involve sensitive topics, such as illicit drug use, sexual behaviors, or political dissent [4]. When faced with such questions, respondents may exhibit protective behaviors due to fears of disclosure and social judgment. These behaviors include direct refusal to participate (unit nonresponse), skipping specific questions (item nonresponse), or providing socially desirable answers. For example, populations at high risk for sexually transmitted infections, such as sex workers, injecting drug users, or men who have sex with men (MSM), often avoid responding to questions about highly sensitive or illegal behaviors, thereby concealing their health conditions [5,6,7]. In these settings, supplementing ego-level responses with information about social connections provides an opportunity to access broader population signals without relying solely on direct self-disclosure. Such measurement issues can cause systematic errors in estimates, which may misinform policy, distort scientific understanding, and ultimately undermine evidence-based decision-making [8,9].

To address these challenges, several indirect questioning methods have been developed. Classical examples include the Randomized Response Technique (RRT) [10] and the Item Count Technique (ICT) [11,12]. In RRT, respondents follow a simple randomization rule so that their answers remain confidential, allowing them to deny revealing sensitive information while still contributing to accurate group estimates. Such designs can also be implemented anonymously to further protect privacy [13,14]. Building on these ideas, the Network Scale-Up Method (NSUM) [15,16] asks respondents to report how many of their peers (alters) belong to a target group, thereby enabling indirect estimation of hidden populations. Many studies have extended NSUM to account for degree heterogeneity, social visibility, and non-random mixing, giving rise to generalized scale-up estimators [17,18,19]. While effective for population size estimation, these methods typically rely on additional assumptions, external information, or auxiliary samples, and often involve complex survey designs with limited statistical efficiency [20,21]. These trade-offs motivate the development of simpler yet robust alternatives for network-based inference.

An alternative approach utilizes the structure of social networks, building on the observation that respondents are often more willing to report on their peers than to disclose their own sensitive attributes [22,23]. Unlike scale-up methods that target population size, this line of work focuses on estimating population proportions from ego-centric samples. This innovative idea was first developed and implemented in the context of Respondent-Driven Sampling (RDS) [24,25]. By exploiting ego-centric network information, the inclusion probability of each ego being reported can be analytically derived, and the asymptotically unbiased estimator $P_{lu}$ [26] was proposed to estimate population proportions in hidden populations [27]. Simulation studies on real-world networks as well as field applications among hard-to-reach populations have shown that $P_{lu}$ substantially outperforms other RDS estimators in terms of both bias and efficiency [28,29,30].

Building on this idea, the Ego-Centric Sampling Method (ECM) [31] collects information through respondents’ ego-centric networks to infer sensitive attributes indirectly. In this design, a representative sample of egos is drawn from the population. Each ego reports the size of their personal network (degree) and the number of peers who possess a specific sensitive attribute (for example, attribute *A*). Instead of asking egos to disclose their own attributes, ECM makes use of their knowledge about peers to estimate population-level proportions. This indirect approach protects respondents’ privacy while retaining the statistical rigor of standard sampling, making it a simple and effective framework for studying sensitive attributes in social and behavioral research.

However, the conventional ECM estimator is unbiased only under the assumption of a homogeneous network, where node degrees are uniform and uncorrelated with attributes. This assumption often fails in heterogeneous networks that exhibit strong degree–attribute correlation. Specifically, if members of one group (for example, attribute *A*) are more active, meaning they have a higher average degree, they are more likely to be reported and thus included in the ego-centric sample once any of their peers is selected. The conventional ECM estimator does not adequately correct for this overrepresentation caused by differences in node activity, which is measured by the activity ratio (AR). As a result, it produces a structural bias that does not diminish with increasing sample size. To date, no general correction for this bias has been established.

To address this limitation, we propose the Activity Ratio Corrected ECM estimator (${\mathrm{ECM}}_{\mathrm{ac}}$). The key idea of ${\mathrm{ECM}}_{\mathrm{ac}}$ is to use the reciprocity property of undirected networks to correct for the imbalance in node activity. Instead of directly estimating the overall population proportion of nodes with attribute *A*, the method focuses on the probabilities of connections between groups (for example, links between *A* and *B* nodes). These edge-based quantities can be measured directly from the ego-centric sample, allowing ${\mathrm{ECM}}_{\mathrm{ac}}$ to obtain an unbiased estimate of P(A) even when node degree and attribute are correlated. This correction substantially improves estimation accuracy in heterogeneous networks where the conventional ECM tends to be biased.

The remainder of this paper is organized as follows. Section 2 details the theoretical framework of the ${\mathrm{ECM}}_{\mathrm{ac}}$ method. We then describe our experimental design, present the simulation and empirical results, and conduct a systematic sensitivity analysis. The paper concludes with a summary of our findings and discusses future research directions.

## 2. Estimation Framework

This section develops the theoretical foundation for our work. We first analyze the statistical properties of the conventional ECM estimator to derive the source of its bias, and then introduce our adjusted estimator, ${\mathrm{ECM}}_{\mathrm{ac}}$, as a direct correction.

### 2.1. Notation and Model Specification

Let G=(V,E) be an undirected simple graph, where $V=\{v_{1},v_{2},\ldots ,v_{N}\}$ is the set of nodes and E⊆V×V is the set of edges. Undirectedness implies $e_{ij}=e_{ji}\in \left\{0,1\right\}$, where $e_{ij}=1$ indicates that an edge exists between nodes *i* and *j*. Each node *i* carries a binary attribute $a_{i}\in \left\{0,1\right\}$, where $a_{i}=1$ denotes nodes belonging to attribute class *A*, and $a_{i}=0$ denotes nodes belonging to attribute class *B*. The degree of node *i* and its decomposition by neighbor attributes are defined as

(1) $$k_{i}=\sum_{j=1}^{N}e_{ij},\;k_{i}^{A}=\sum_{j=1}^{N}e_{ij}\;a_{j},\;k_{i}^{B}=k_{i}-k_{i}^{A}.$$

The quantity of interest is the population proportion of nodes with attribute *A*, which is the main target of estimation:

(2) $$P\left(A\right)=\frac{1}{N}\sum_{i=1}^{N}a_{i}.$$

Let $A=\{i:a_{i}=1\}$ and $B=\{i:a_{i}=0\}$ with sizes $N_{A}=\left|A\right|$ and $N_{B}=\left|B\right|$. The group mean degrees and the activity ratio are defined as

(3) $${\bar{k}}_{A}=\frac{1}{N_{A}}\sum_{i\in A}k_{i},\;{\bar{k}}_{B}=\frac{1}{N_{B}}\sum_{i\in B}k_{i},\;AR=\frac{{\bar{k}}_{A}}{{\bar{k}}_{B}}.$$

### 2.2. Observables from Ego-Centric Sampling

**Figure 1:**
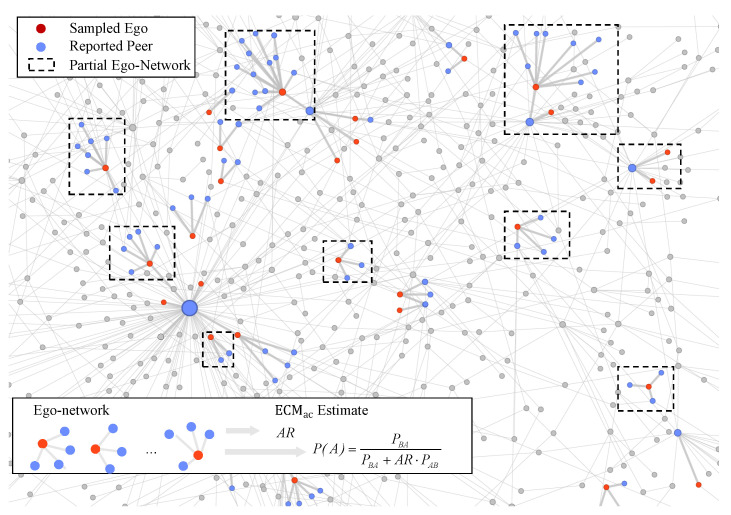
Workflow of the Ego-Centric Sampling Method (ECM) and the activity-corrected estimator ${\mathrm{ECM}}_{\mathrm{ac}}$. A random sample of respondents (sampled egos) is drawn from the full network population. Each sampled ego then reports on the attributes of their direct connections (reported peers), providing data on their partial ego-network. As illustrated in the inset, this local data is aggregated to compute the activity ratio (AR) and connection probabilities required by the ${\mathrm{ECM}}_{\mathrm{ac}}$ estimator for P(A).

Ego-centric sampling collects local network information in two stages. First, a set of *S* respondents (egos) is drawn from the population, typically by simple random sampling. Second, each sampled ego i∈S reports their degree and the number of peers in each attribute class, recorded as $\left(k_{i},\;k_{i}^{A},\;k_{i}^{B}\right)$, as illustrated in Figure 1.

We partition the sample according to the respondent’s attribute:

(4) $$S_{A}=\left\{i\in S:a_{i}=1\right\},\;S_{B}=\left\{i\in S:a_{i}=0\right\},$$

and define the total degrees within each group as

(5) $$s_{A}=\sum_{i\in S_{A}}k_{i},\;s_{B}=\sum_{i\in S_{B}}k_{i}.$$

The cross-group neighbor counts, aggregated from the respondents’ side, are defined as

(6) $$m_{AB}=\sum_{i\in S_{A}}k_{i}^{B},\;m_{BA}=\sum_{i\in S_{B}}k_{i}^{A}.$$

Based on these observations, we define ${\hat{P}}_{AB}^{\left(S\right)}$ as the probability that a neighbor of an *A*-node belongs to *B*, and ${\hat{P}}_{BA}^{\left(S\right)}$ as its reverse counterpart. The sample activity ratio is denoted by $\hat{AR}$:

(7) $${\hat{P}}_{AB}^{\left(S\right)}=\frac{m_{AB}}{s_{A}},\;{\hat{P}}_{BA}^{\left(S\right)}=\frac{m_{BA}}{s_{B}},\;\hat{AR}=\frac{s_{A}/\left|S_{A}\right|}{s_{B}/\left|S_{B}\right|}.$$

### 2.3. ECM Estimator

The traditional ECM estimator [31] begins with a decomposition by degree strata. Let $n_{k}$ be the number of nodes with degree *k*, and p(A∣k) be the conditional probability that a node of degree *k* belongs to class *A*. The total number of edges emanating from *A*-nodes of degree *k* is given by

(8) $$\sum_{i\in \{i|k_{i}=k\}}k_{i}^{A}=n_{k}\cdot p\left(A|k\right)\cdot k.$$

The expected number of nodes with attribute *A* in the population can therefore be written as

(9) $$E\left(N_{A}\right)=\sum_{k=1}^{k_{\max}}p\left(A|k\right)\;n_{k},$$

which implies that the population proportion is $P\left(A\right)=N^{-1}\sum_{k}n_{k}p\left(A|k\right)$. Combining this with (8), an idealized (theoretical) form of the estimator can be expressed as

(10) $${\hat{P}}_{\mathrm{ideal}}\left(A\right)=\frac{\sum_{k=1}^{k_{\max}}\sum_{i\in \{i|k_{i}=k\}}\frac{k_{i}^{A}}{k}}{N}.$$

In studies involving hidden or hard-to-reach populations, the full network is typically unobservable. Therefore, the sample mean is used as a practical substitute for the corresponding population quantity, yielding the ECM estimator:

(11) $${\hat{P}}_{\mathrm{ECM}}\left(A\right)=\frac{1}{\left|S\right|}\sum_{i\in S}\frac{k_{i}^{A}}{k_{i}}.$$

ECM assumes that node degree is independent of attribute type. Under this condition, the unweighted average across sampled egos’ proportions of *A*-type neighbors equals the population proportion P(A). When groups differ in their mean degree, the more active group becomes overrepresented and the ECM estimator is biased, which motivates the adjusted estimator ${\mathrm{ECM}}_{\mathrm{ac}}$.

### 2.4. ${\mathrm{ECM}}_{\mathrm{ac}}$ Based on Reciprocity

We derive ${\mathrm{ECM}}_{\mathrm{ac}}$ from two basic equalities that hold in the undirected graph *G*. Because the graph is undirected, the number of cross-group edges counted from *A* to *B* must equal the number counted from *B* to *A*. This structural equality is referred to as reciprocity:

(12) $$E_{AB}=E_{BA}.$$

Let $P_{AB}$ and $P_{BA}$ denote the probabilities that an edge attached to an *A*-node or a *B*-node, respectively, connects to the other group. These are defined as

(13) $$P_{AB}=\frac{\sum_{i\in A}k_{i}^{B}}{\sum_{i\in A}k_{i}},\;P_{BA}=\frac{\sum_{j\in B}k_{j}^{A}}{\sum_{j\in B}k_{j}}.$$

By definition, the number of cross-group edges can be expressed in two equivalent forms:

(14) $$E_{AB}=N_{A}\;{\bar{k}}_{A}\;P_{AB},\;E_{BA}=N_{B}\;{\bar{k}}_{B}\;P_{BA}.$$

Starting from this relation, $N_{A}{\bar{k}}_{A}P_{AB}=N_{B}{\bar{k}}_{B}P_{BA}$, we divide both sides by $N{\bar{k}}_{B}$ and use $P\left(A\right)=N_{A}/N$, $1-P\left(A\right)=N_{B}/N$, and $AR={\bar{k}}_{A}/{\bar{k}}_{B}$ to obtain:

(15) $$P\left(A\right)\;AR\;P_{AB}=\left(1-P\left(A\right)\right)\;P_{BA}.$$

From Equation (15), we derive a population-level relationship that forms the theoretical basis for the ${\mathrm{ECM}}_{\mathrm{ac}}$ estimator: Expanding the right side of Equation (15), we get $P\left(A\right)\cdot AR\cdot P_{AB}=P_{BA}-P\left(A\right)\cdot P_{BA}$. Rearranging the terms to group P(A) yields $P\left(A\right)\left(AR\cdot P_{AB}+P_{BA}\right)=P_{BA}$.

From this, we derive the population-level relationship that forms the theoretical basis for the ${\mathrm{ECM}}_{\mathrm{ac}}$ estimator:

(16) $$P\left(A\right)=\frac{P_{BA}}{P_{BA}+AR\cdot P_{AB}}.$$

The corresponding sample-based estimator is obtained by the plug-in principle, substituting the population quantities in Equation (16) with their empirical counterparts:

(17) $${\hat{P}}_{\mathrm{ac}}\left(A\right)=\frac{{\hat{P}}_{BA}^{\left(S\right)}}{{\hat{P}}_{BA}^{\left(S\right)}+\hat{AR}\cdot {\hat{P}}_{AB}^{\left(S\right)}}.$$

Using Equation (7), the estimator can be written directly in terms of the observed sample counts:

(18) $${\hat{P}}_{\mathrm{ac}}\left(A\right)=\frac{m_{BA}s_{A}}{m_{BA}s_{A}+\hat{AR}\;m_{AB}s_{B}}.$$

### 2.5. Variance Estimation

The analytical variance of network-based estimators is typically intractable. It depends on complex networks features such as topology, degree heterogeneity, and inter-node dependence. To obtain empirical variance estimates and construct confidence intervals for ${\mathrm{ECM}}_{\mathrm{ac}}$, we adopt a nonparametric bootstrap approach.

The bootstrap procedure proceeds as follows:

(1) Draw a bootstrap replicate by resampling ego-centric networks (each ego together with its reported peers) with replacement, and denote the resulting bootstrap sample as $B_{b}$;
(2) Based on $B_{b}$, compute the corresponding estimator ${\hat{P}}^{*(b)}\left(A\right)$ using ${\mathrm{ECM}}_{\mathrm{ac}}$;
(3) Repeat steps (1)–(2) for b=1,2,…,B, obtaining a set of bootstrap estimates:
  (19) $$\{{\hat{P}}^{*(1)}\left(A\right),{\hat{P}}^{*(2)}\left(A\right),\ldots ,{\hat{P}}^{*(B)}\left(A\right)\}.$$
(4) Sort these estimates in ascending order, and construct the (1−α) percentile confidence interval as
  (20) $${\mathrm{CI}}_{1-\alpha }=\left[{\hat{P}}^{*(\lceil \alpha B/2\rceil )}\left(A\right),{\hat{P}}^{*(\lceil (1-\alpha /2)B\rceil )}\left(A\right)\right].$$

Since different resampling schemes correspond to different assumptions about dependence in the data, we consider three bootstrap designs to reflect multiple sources of uncertainty:

BS-Ego assumes that ego-centric networks are independent sampling units. It captures between-ego variation and reflects uncertainty due to the limited number of egos.

BS-Tree adds within-ego resampling, treating each ego’s reported peers as nested observations. This design accounts for additional variability introduced by the hierarchical (ego–peer) structure of ego-centric data [32].

BS-Pool treats all ego–peer pairs as exchangeable edges and resamples them directly. It reflects uncertainty arising from the random formation of connections rather than from the selection of egos.

**Figure 2:**
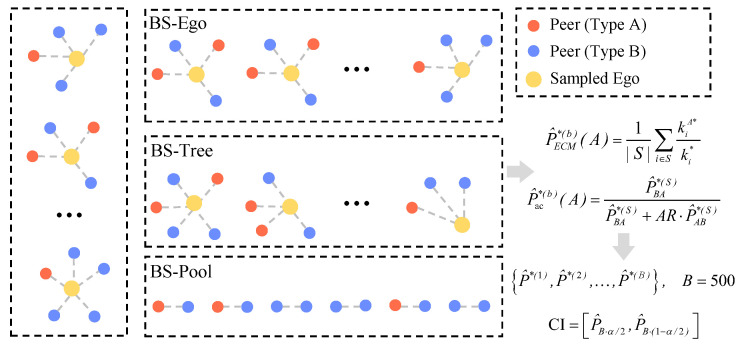
Bootstrap workflow for ${\mathrm{ECM}}_{\mathrm{ac}}$. Starting from the original ego-centric sample, bootstrap datasets are generated under three schemes, and for each dataset ${\hat{P}}_{\mathrm{ac}}\left(A\right)$ is recomputed; percentile confidence intervals are then obtained from the empirical quantiles of the replicates. BS-Ego resamples whole egos together with their reported peers and treats egos as independent units. BS-Tree resamples egos and, within each sampled ego, resamples the reported peers to capture within-ego dependence, whereas BS-Pool resamples all reported ego–peer pairs as exchangeable edges.

These three schemes differ only in how resampled ego-centric datasets are constructed. Together, they provide complementary perspectives on estimator variability under distinct dependence assumptions. BS-Ego assumes independent egos, BS-Tree allows dependent peers within the same ego, and BS-Pool assumes independence only at the edge level. A workflow overview is shown in Figure 2, and detailed algorithmic descriptions for each scheme are provided in Appendix A.

## 3. Experimental Design

### 3.1. Synthetic and Real-World Networks

We rigorously evaluate the performance of ${\mathrm{ECM}}_{\mathrm{ac}}$ against the conventional ECM using a comprehensive framework spanning both synthetic and real-world networks.

Synthetic Networks. We generated Erdős–Rényi (ER) and Barabási–Albert (BA) networks of 10,000 nodes to represent homogeneous and heterogeneous degree distributions, respectively. Nodes were assigned a binary attribute (*A* or *B*) to achieve target proportions P(A)∈{0.1,0.2,0.3,0.4}. Within these networks, we systematically control four key structural properties:

(1) *Density* (Ψ): quantifies the overall connectivity of a network [33] and is calculated as
  (21) $$\Psi =\frac{2E}{N(N-1)},$$
  where *E* is the number of edges, and *N* is the number of nodes in the network.
(2) *Average Clustering Coefficient* ($C_{\mathrm{avg}}$): is a measure of how nodes tend to cluster together [34]. For each node *i*, the local clustering coefficient is defined as
  (22) $$C_{i}=\frac{2\Delta_{i}}{k_{i}\left(k_{i}-1\right)},$$
  where $k_{i}$ is the degree of node *i*, and $\Delta_{i}$ is the number of triangles that node *i* forms with its peers. The overall average clustering coefficient is then the mean of all individual $C_{i}$:
  (23) $$C_{\mathrm{avg}}=\frac{1}{N}\sum_{i=1}^{N}C_{i}.$$
(3) *Homophily* (*H*): quantifies the extent to which nodes prefer connections within their own group rather than across groups. Let $S_{AA}^{*}$ denote the proportion of A→A links among all links originating from *A*-nodes [35,36]. The definition of *H* is as follows:
  (24) $$S_{AA}^{*}=H+\left(1-H\right)P_{A},$$
  when H=1, all *A*-nodes only connect to other *A*-nodes (perfect assortative mixing); when H=0, *A*-nodes connect to others proportionally to group sizes (random mixing); intermediate values 0<H<1 indicate partial within-group preference. Negative values (H<0) correspond to disassortative mixing, i.e., a tendency to connect across groups [37].
(4) *Activity Ratio* (AR): is set to values in the range [0.5,2.5] by swapping attributes between high- and low-degree nodes to induce specific levels of degree–attribute correlation, while preserving both the network topology and marginal attribute counts [38].

Each network property was systematically tuned by modifying one factor at a time from its baseline BA configuration, ensuring that all other metrics remained stable within 1%. Specifically, Ψ was increased by randomly adding edges, $C_{\mathrm{avg}}$ was adjusted through targeted triad closure rewiring, and *H* was tuned by randomly reconnecting cross-group or within-group links. The controlled parameter ranges were Ψ∈[0.002,0.10] (step t = 0.002), $C_{\mathrm{avg}}\in \left[0.001,0.03\right]$ (t = 0.001), H∈[−0.30,0.25] (t = 0.05), and AR∈[0.5,2.5] (t = 0.1).

### 3.2. Real-World Networks

To evaluate performance in practical settings, we selected six diverse real-world networks spanning both molecular and social domains, with broad variation in size, semantics, and degree heterogeneity. Each dataset provides node-level categorical labels and undirected connectivity from publicly available repositories.

AIDS: Derived from the Network Repository [39], representing molecular graphs where nodes are atoms and edges are chemical bonds (single, double, or aromatic).

PTC: From the Predictive Toxicology Challenge dataset [39], containing chemical compounds for carcinogenicity prediction, generated using the Chemistry Development Kit (v1.4).

Git: A GitHub developer network collected from the public API [40], where users are connected via mutual following relationships. Node metadata include location, employer, and starred repositories.

Flickr: An online community network [39], with users linked by shared interests or mutual following. Labels identify user groups or communities.

Tox: From the Tox21 toxicity database [39], comprising molecular graphs with atoms as nodes and bonds as edges. Labels correspond to atom types.

Twitter: A social interaction network from the Network Repository [39], where users are connected through interaction edges. Labels are derived from dominant textual themes (e.g., “love” and “sleep”).

For all datasets, the *A*-class is defined as the less frequent label to mimic imbalance in real populations. Summary statistics are reported in Table 1.

### 3.3. Sampling and Estimation Procedure

To implement our simulation protocol, we first draw a simple random sample of egos (10% of total nodes) without replacement from each network. For each sampled ego, we then collect neighborhood information using one of four distinct sampling strategies: full reporting (F); partial random sampling of 5 (P5) or 10 (P10) peers; and weighted sampling (W), where 10 peers are drawn with probabilities inversely proportional to their degrees.

### 3.4. Evaluation Metrics

Estimator performance is evaluated using Bias, Standard Deviation (SD), and Root Mean Squared Error (RMSE) for point accuracy, and empirical coverage rates for interval reliability. We also report the percentage of trials where an estimator achieves the lowest error ($P_{\mathrm{best}}$).

### 3.5. Bootstrap Confidence Intervals

We evaluate interval estimation through a simulation-based bootstrap experiment. For each network configuration with a given P(A), 10% of nodes are randomly sampled to form ego-centric samples. Using the bootstrap procedure in the Variance Estimation, 90% and 95% confidence intervals are constructed from B=1000 resamples, and the process is repeated R=1000 times to estimate empirical coverage. All simulations follow the same sampling settings described above.

## 4. Results

### 4.1. Performance on Synthetic Networks

**Figure 3:**
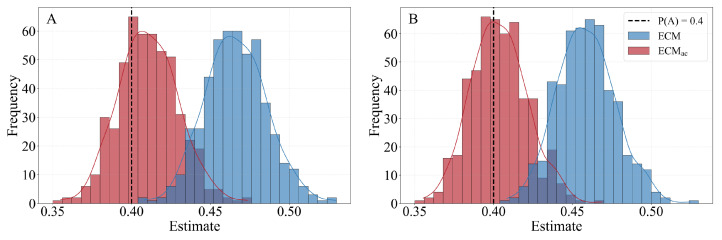
Estimate distributions for ECM and ${\mathrm{ECM}}_{\mathrm{ac}}$ in a BA network with H=0.15, AR=1.3, and a true P(A)=0.40. Under both full reporting (**A**) and partial reporting of 10 peers (**B**), the conventional ECM estimator shows significant bias, with its distribution peaking near 0.475. The proposed ${\mathrm{ECM}}_{\mathrm{ac}}$ estimator, however, remains centered on the true value in both cases.

We begin by examining the fundamental performance of the estimators in scale-free BA networks. Figure 3 illustrates a representative setting with homophily fixed at H=0.15, AR =1.3, and a true population proportion P(A)=0.40. The results clearly show that the conventional ECM estimator is severely biased, with its estimate distribution peaking near 0.475, a substantial overestimation of the benchmark. In contrast, the ${\mathrm{ECM}}_{\mathrm{ac}}$ distribution is centered on the true value, demonstrating its ability to correct for degree–attribute bias.

This finding is quantitatively substantiated in Table 2 and Table 3. Table 2 summarizes results across both BA and ER network models, showing that ${\mathrm{ECM}}_{\mathrm{ac}}$ consistently achieves lower bias and RMSE than ECM whenever AR ≠1. For instance, in BA networks with AR=1.5 and P(A)=0.1, ${\mathrm{ECM}}_{\mathrm{ac}}$ reduces the RMSE by more than 70% relative to ECM. As predicted by theory, when AR=1 the two estimators perform identically. Table 3 further demonstrates that ${\mathrm{ECM}}_{\mathrm{ac}}$’s advantage is robust across different sampling protocols (F, P5, P10, and W). Across all cases, ${\mathrm{ECM}}_{\mathrm{ac}}$ not only yields the lowest RMSE but also attains the highest winning percentage ($P_{\mathrm{best}}$), underscoring its practical utility.

### 4.2. Performance on Real-World Networks

Our empirical analysis across six diverse real-world networks further validates the effectiveness of ${\mathrm{ECM}}_{\mathrm{ac}}$, showing robustness in both molecular and social settings. We assess two complementary aspects: (i) how estimates converge as the sample fraction grows, and (ii) how bias distributions behave under different sampling strategies (F, P, W). Across all networks, ${\mathrm{ECM}}_{\mathrm{ac}}$ remains centered on the true proportion and exhibits stable performance, whereas ECM shows systematic deviations whenever AR departs from one.

**Figure 4:**
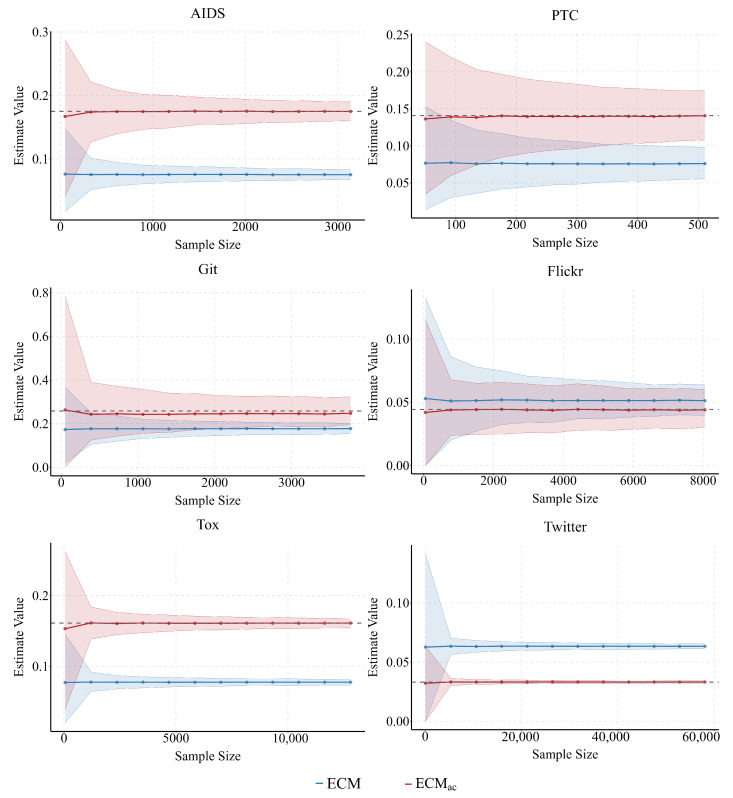
Estimates of ECM and ${\mathrm{ECM}}_{\mathrm{ac}}$ across six real networks as sample size increases. Solid lines show the mean estimates and shaded bands the 90% empirical confidence interval (5th–95th percentile); ${\mathrm{ECM}}_{\mathrm{ac}}$ consistently tracks the true proportion (grey dashed line), while ECM converges to biased values in the presence of degree–attribute imbalance.

The results show that ${\mathrm{ECM}}_{\mathrm{ac}}$ consistently aligns more closely with the true P(A) across all networks, whereas the conventional ECM exhibits systematic deviations whenever AR departs from one (see Figure 4).

For example, in networks with AR<1 such as AIDS (AR ≈0.54), Git (AR ≈0.49), and Tox (AR ≈0.58), the conventional ECM persistently underestimates P(A). In Git, this downward bias is particularly severe, with ECM converging to an estimate nearly 30% below the true value, ${\mathrm{ECM}}_{\mathrm{ac}}$ effectively eliminates this discrepancy. Conversely, in networks with AR>1, such as Twitter (AR ≈1.64), ECM systematically overestimates the population proportion. Across all cases, ${\mathrm{ECM}}_{\mathrm{ac}}$ produces estimates centered on the true value, underscoring that correcting for degree–attribute correlation is essential for accurate inference in empirical networks.

**Figure 5:**
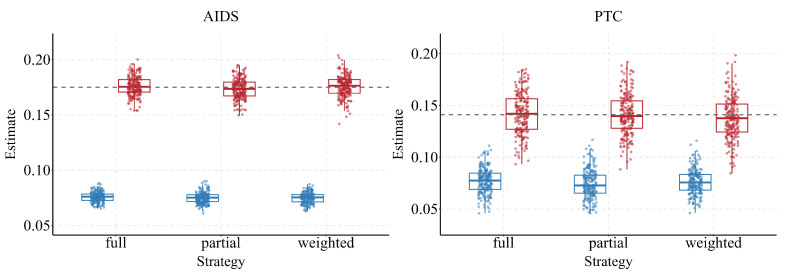

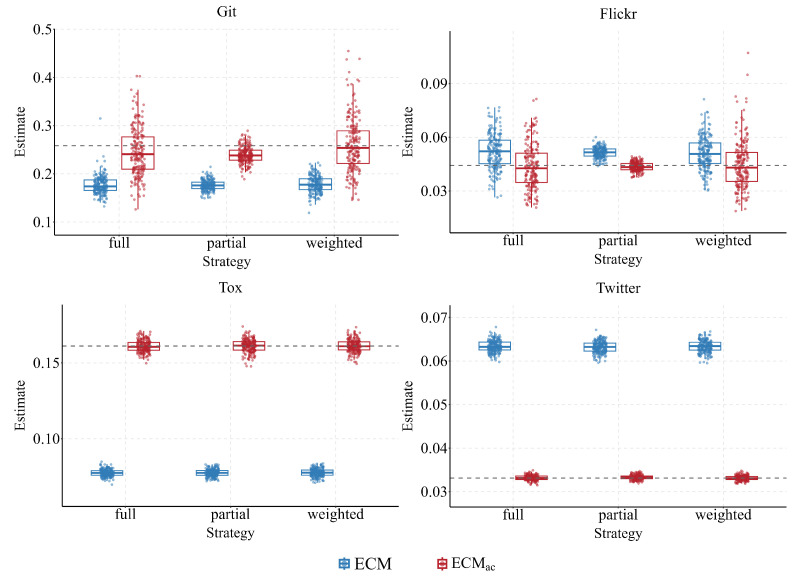
Distributions of ECM and ${\mathrm{ECM}}_{\mathrm{ac}}$ estimates under full, partial, and weighted ego-centric sampling across six real networks. The grey dashed line is the true P(A). Dots are replicate estimates and boxplots summarize their spread. Across networks and strategies, ${\mathrm{ECM}}_{\mathrm{ac}}$ stays close to the truth and reduces bias relative to ECM.

Furthermore, ${\mathrm{ECM}}_{\mathrm{ac}}$ demonstrates strong robustness across different sampling strategies (F, P, W), as shown in Figure 5. This consistency is evident across all six networks. For instance, in Twitter (AR ≈1.64), the distribution of ${\mathrm{ECM}}_{\mathrm{ac}}$ estimates remains centered near the true value (P(A)≈0.035) under all strategies, whereas ECM consistently overestimates the proportion by approximately +0.01 to +0.015. A symmetric pattern is observed in networks with AR<1, such as AIDS (AR ≈0.54) and Git (AR ≈0.49), where ECM exhibits a downward bias of about −0.03 to −0.05, while ${\mathrm{ECM}}_{\mathrm{ac}}$ stays closely aligned with the true benchmark.

In summary, empirical evidence from these diverse real-world networks confirms that ${\mathrm{ECM}}_{\mathrm{ac}}$ substantially reduces estimation bias relative to ECM, particularly when AR deviates substantially from one. These improvements establish ${\mathrm{ECM}}_{\mathrm{ac}}$ as a reliable and practical estimator for network-based attribute inference in real-world applications.

For completeness, we provide an additional supplementary comparison with two representative baseline approaches (RDS-II and NSUM) on real-world networks in Appendix B. Because these methods rely on different sampling mechanisms and target estimands, the results are presented for reference only.

### 4.3. Bootstrap Coverage Rate

To evaluate interval reliability under controlled structural conditions, we conduct bootstrap experiments on synthetic networks generated by the BA model. We vary AR from 0.8 to 1.8 and P(A) from 0.10 to 0.40, and construct 90% and 95% percentile confidence intervals using three resampling schemes.

Table 4 and Table 5 report the empirical coverage. At the 95% level, BS-Ego is consistently closest to the nominal target across the (AR,P(A)) grid, whereas BS-Tree is conservative and BS-Pool exhibits systematic under-coverage.

BS-Ego provides the best-calibrated inference for 95% confidence intervals, while BS-Tree only yields acceptable 90% coverage at the cost of wider intervals. This difference follows from the data-generating mechanism of ego-centric sampling: egos are the primary sampling units, with peer reports clustered within each ego-network. By resampling intact ego-networks, BS-Ego preserves this dependence structure and accurately reflects sampling variability, whereas BS-Tree inflates variability by resampling peers and BS-Pool ignores ego-level clustering.

## 5. Sensitivity Analysis

We now turn to a systematic analysis of how estimator performance varies with key network and attribute parameters. All supporting tables for the figures in this section are included in Supporting Information Tables S1–S5.

### 5.1. Population Proportion P(A)

**Figure 6:**
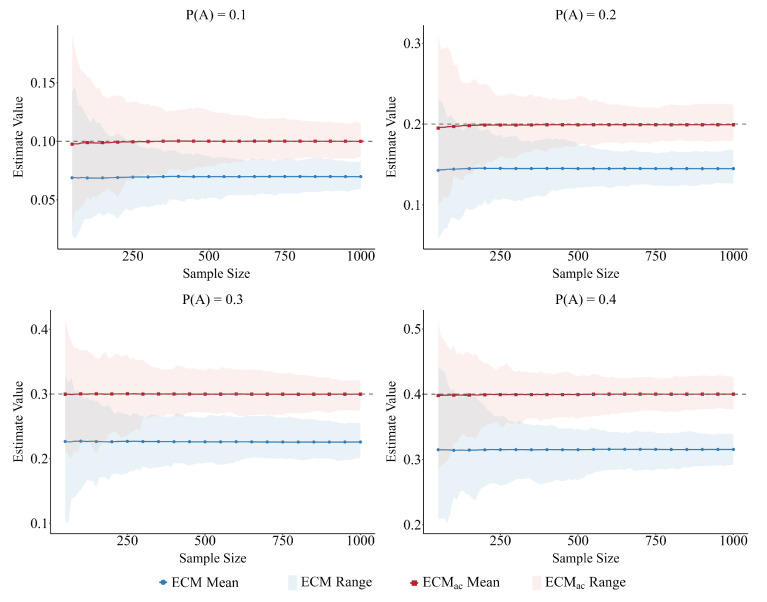
Effect of P(A) on estimator accuracy in BA networks under fixed sampling settings. Curves show mean estimates and shaded bands denote 95% confidence intervals. As P(A) increases from 0.1 to 0.4, precision improves for both estimators, and ECM exhibits a persistent downward bias that is most pronounced at small P(A), while ${\mathrm{ECM}}_{\mathrm{ac}}$ remains centered on the true value across all levels.

We evaluate the effect of the population proportion on estimator accuracy using BA networks under fixed sampling settings, varying P(A) from 0.1 to 0.4 while keeping all other parameters constant. As shown in Figure 6, increasing P(A) leads both estimators to become more precise, as indicated by narrower 95% confidence intervals. Across all tested conditions, however, ${\mathrm{ECM}}_{\mathrm{ac}}$ remains centered on the true population proportion, whereas ECM exhibits a persistent downward bias that becomes more pronounced when P(A) is small. For example, when P(A)=0.1, the mean estimate of ECM is approximately 0.07, underestimating the true value by nearly 30%, while ${\mathrm{ECM}}_{\mathrm{ac}}$ yields a mean of 0.10, closely matching the benchmark. When P(A) increases to 0.4, the mean estimate of ECM remains around 0.32, which is still about 20% below the true value, whereas ${\mathrm{ECM}}_{\mathrm{ac}}$ stays nearly unbiased.

This demonstrates that even when sampling uncertainty decreases with larger P(A), the degree–attribute bias inherent in ECM persists, while ${\mathrm{ECM}}_{\mathrm{ac}}$ effectively eliminates it.

### 5.2. Activity Ratio (AR)

**Figure 7:**
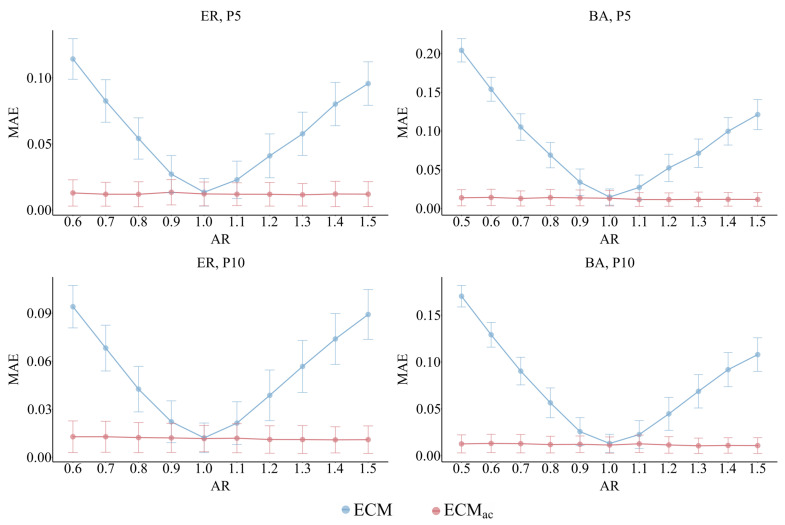
Sensitivity to AR, illustrating the MAE of ECM and ${\mathrm{ECM}}_{\mathrm{ac}}$ in ER and BA networks under P5 and P10 sampling. ECM shows a pronounced U-shaped MAE as AR departs from 1, whereas ${\mathrm{ECM}}_{\mathrm{ac}}$ remains nearly flat with substantially lower error (largest gains when AR ≤ 0.7 or ≥ 1.3). Error bars denote 95% confidence intervals.

We examine the impact of degree–attribute imbalance by varying AR in both ER and BA networks while keeping other parameters fixed. Figure 7 presents results under the P5 and P10 sampling strategies, which represent realistic partial-reporting scenarios where egos disclose only a limited number of peers. The omitted full-reporting (F) and weighted (W) schemes exhibit nearly identical bias patterns, differing only in the overall width of confidence intervals rather than in systematic trends, with all showing the same direction of bias relative to AR.

Across both network types, ECM displays a distinct U-shaped error curve as AR deviates from one. In BA networks, the MAE of ECM rises sharply from approximately 0.03 at AR=1.0 to over 0.20 at AR=0.5 and AR=1.5. By contrast, ${\mathrm{ECM}}_{\mathrm{ac}}$ remains almost flat across all tested AR values, with MAE typically below 0.02 even under strong imbalance. This corresponds to an 85–90% reduction in MAE relative to ECM when AR≤0.7 or AR≥1.3.

These results demonstrate that accounting for degree–attribute correlation is essential for reducing activity-induced bias, particularly under realistic partial-reporting conditions.

### 5.3. Network Density and Clustering

**Figure 8:**
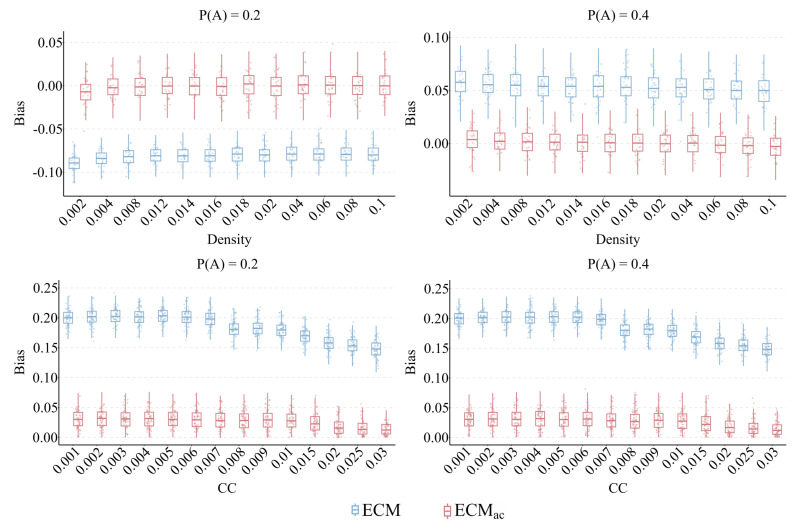
Sensitivity to network density (Ψ) and clustering ($C_{\mathrm{avg}}$). This plot shows the MAE of ECM and ${\mathrm{ECM}}_{\mathrm{ac}}$ as Ψ and $C_{\mathrm{avg}}$ increase. Although higher connectivity slightly impacts both estimators, ECM’s error escalates significantly. In contrast, ${\mathrm{ECM}}_{\mathrm{ac}}$ demonstrates strong robustness, maintaining a consistently low and stable error profile. Shaded areas represent 95% confidence intervals.

We further investigate how structural connectivity influences estimator performance by varying network density (Ψ) and average clustering coefficient ($C_{\mathrm{avg}}$) while keeping AR and P(A) fixed. As shown in Figure 8, increasing either density or clustering slightly reduces estimation accuracy for both estimators. However, the deterioration of ${\mathrm{ECM}}_{\mathrm{ac}}$ is much less pronounced. When network density increases from 0.002 to 0.10, the median bias of ECM rises gradually from approximately 0.03 to 0.08, whereas ${\mathrm{ECM}}_{\mathrm{ac}}$ remains close to zero, with deviations below 0.01. A similar pattern is observed when $C_{\mathrm{avg}}$ reaches 0.03, where ECM’s bias stabilizes around 0.18 to 0.20, roughly six times higher than that of ${\mathrm{ECM}}_{\mathrm{ac}}$, whose bias remains below 0.03.

These results indicate that denser or more clustered networks amplify the bias arising from degree–attribute correlation, but the correction introduced by ${\mathrm{ECM}}_{\mathrm{ac}}$ effectively stabilizes estimation performance across a wide range of structural conditions.

### 5.4. Combined Effects of AR and H

Building upon the previous single-factor analyses, we further examine how the interaction between degree–attribute imbalance and assortative mixing jointly shapes estimator bias. Specifically, we vary both AR and the homophily (*H*) to capture their combined influence on estimator performance.

**Figure 9:**
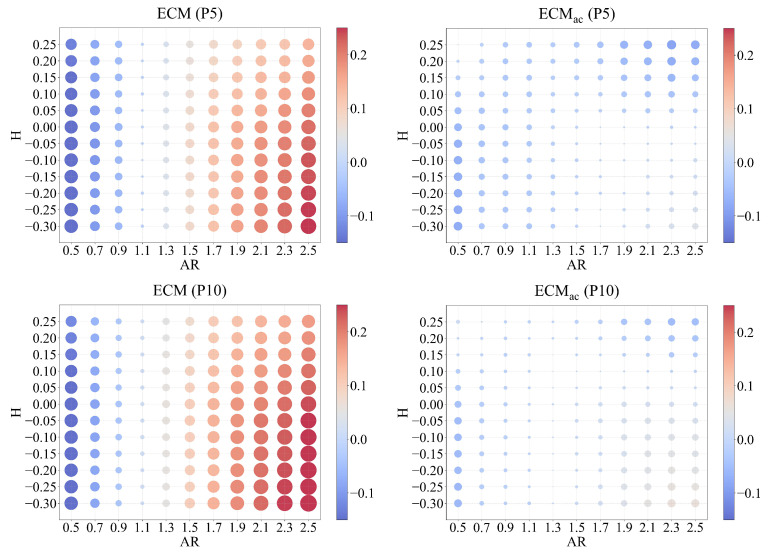
Joint effect of AR and *H*. Estimation error of ECM and ${\mathrm{ECM}}_{\mathrm{ac}}$ across the parameter space defined by AR and *H*, with bubble size encoding error magnitude. ECM’s error shows a strong, complex dependency on both factors, particularly at extreme AR values and under disassortative mixing (H<0). In contrast, ${\mathrm{ECM}}_{\mathrm{ac}}$ displays a uniformly low error surface, highlighting its robustness to the interaction between network structure and degree–attribute correlations.

Figure 9 illustrates these joint effects, showing that the bias field of ${\mathrm{ECM}}_{\mathrm{ac}}$ remains stable across the full range of structural conditions. For ECM, bias patterns form a pronounced gradient across the (AR,H) plane, where negative homophily (H<0) and extreme AR values (AR>2 or AR<0.7) yield the largest deviations, with estimation errors reaching about +0.25 or −0.18. When homophily becomes positive, ECM tends to overestimate the true proportion, whereas negative homophily induces underestimation. In contrast, ${\mathrm{ECM}}_{\mathrm{ac}}$ exhibits an almost uniform error surface, with absolute bias remaining below 0.03 under both P5 and P10 sampling.

It is worth noting that in highly assortative networks (H→1), cross-group connection probabilities approach zero ($P_{AB},P_{BA}\to 0$), theoretically risking numerical instability. In practice, this is rare in connected networks and can be addressed by applying a small smoothing factor, as implemented in our bootstrap procedure.

These findings demonstrate that ${\mathrm{ECM}}_{\mathrm{ac}}$ effectively mitigates the compounded bias arising from the coexistence of degree–attribute correlation and homophily.

### 5.5. Combined Effects of AR and P(A)

**Figure 10:**
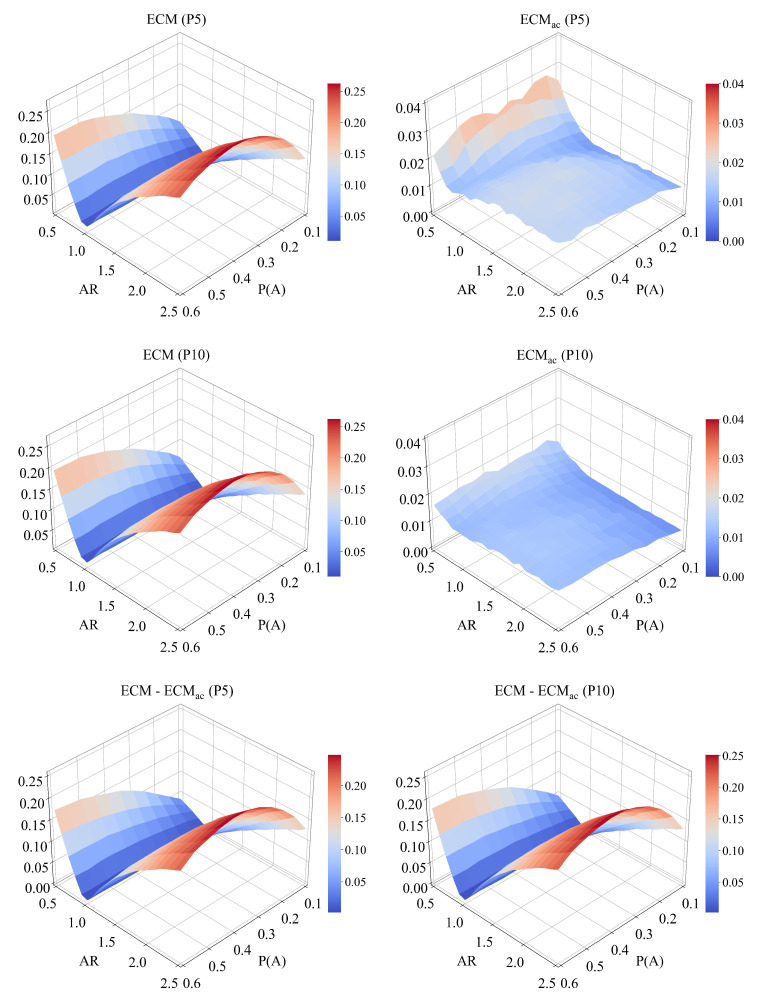
Bias surfaces of ECM and ${\mathrm{ECM}}_{\mathrm{ac}}$ in the AR×P(A) parameter space. The top and middle rows correspond to the P5 and P10 sampling quotas, respectively. ECM’s bias forms a pronounced ridge, escalating as AR deviates from 1 and P(A) increases. In contrast, ${\mathrm{ECM}}_{\mathrm{ac}}$ produces a consistently flat, low-bias surface. The bottom row visualizes the point-wise bias reduction, highlighting that the correction is most effective precisely where ECM is most biased.

To further investigate how degree heterogeneity and group proportion jointly influence estimator performance, we analyze bias across the (AR,P(A)) parameter space under partial reporting. Figure 10 presents the resulting bias surfaces for both P5 and P10 sampling strategies.

For ECM, bias increases sharply when either AR≠1 or P(A) becomes large, forming a pronounced ridge around (AR,P(A))≈(2.5,0.6), where the bias reaches approximately 0.24. Under P10 sampling, the mean bias for ECM is 0.104 (SD = 0.061). In contrast, ${\mathrm{ECM}}_{\mathrm{ac}}$ produces a much flatter bias surface, with values remaining below 0.04 and a mean of 0.026 (SD = 0.016). The lower panels show that local bias reduction can reach 0.25 at (AR,P(A))=(2.5,0.35), confirming the consistent advantage of the activity-corrected estimator.

Smaller sampling strategies (P5) lead to slightly higher overall biases than P10, indicating that including more peers per ego improves estimator stability even when the number of egos is fixed. Overall, these results highlight that ${\mathrm{ECM}}_{\mathrm{ac}}$ remains robust under the combined effects of group imbalance and degree heterogeneity, maintaining low bias across a wide range of network conditions.

## 6. Conclusions

While the conventional ECM estimator relies on degree-based weighting to infer group proportions from ego-centric samples, it often exhibits systematic bias in heterogeneous networks—particularly when node degree is correlated with node attributes. To overcome this limitation, this study introduces the Activity Ratio Corrected ECM estimator (${\mathrm{ECM}}_{\mathrm{ac}}$), which explicitly incorporates degree–attribute correlation through an activity ratio adjustment, thereby enhancing both the adaptability and accuracy of proportion estimation in complex networks.

By leveraging the principle of reciprocity, we reformulated the group proportion estimation problem into an edge-based framework that estimates cross-group connection probabilities ($P_{AB}$ and $P_{BA}$) without requiring global network information. This formulation effectively mitigates the structural bias inherent in the traditional ECM approach and enables unbiased proportion estimation using only locally observed ego-network data.

Simulation experiments demonstrate that ${\mathrm{ECM}}_{\mathrm{ac}}$ consistently reduces estimation bias and lowers RMSE across a broad range of network structures, including variations in density, clustering, and homophily. Sensitivity analyses further show that ${\mathrm{ECM}}_{\mathrm{ac}}$ maintains robustness across different attribute ratios and sampling designs, producing nearly flat bias surfaces and stable performance even under extreme network heterogeneity. Empirical validations on six real-world networks confirm that ${\mathrm{ECM}}_{\mathrm{ac}}$ accurately corrects the overestimation and underestimation observed in traditional ECM.

Overall, the proposed ${\mathrm{ECM}}_{\mathrm{ac}}$ estimator provides a theoretically sound and practically effective framework for attribute estimation in complex social networks. By addressing the key limitations of traditional ECM in heterogeneous environments, this method offers a reliable foundation for privacy-preserving surveys, social network inference, and other applications requiring indirect estimation from local sampling data.

## Figures and Tables

**Table 1:** Overview of real-world network datasets.

Network	Nodes	Edges	Density	Clustering	P(A)	AR
AIDS	31,385	32,390	0.00007	0.005	0.1750	0.54
PTC	5110	54,690	0.00040	0.006	0.1411	0.60
Git	37,700	289,003	0.00041	0.168	0.2583	0.49
Flickr	80,513	5,900,000	0.00182	0.165	0.4420	1.22
Tox	127,998	130,481	0.00001	0.003	0.1611	0.58
Twitter	580,800	1,400,000	0.00001	0.394	0.0331	1.64

**Table 2:** Bias (SD) and RMSE (P^best^) of sample, ECM, and ${\mathrm{ECM}}_{\mathrm{ac}}$ estimators under varying AR in synthetic networks.

AR	Bias (SD)			RMSE (P^best^)		
	**Sample**	**ECM**	** ${\mathrm{ECM}}_{\mathrm{ac}}$ **	**Sample**	**ECM**	** ${\mathrm{ECM}}_{\mathrm{ac}}$ **
**BA network,** P(A)=0.1						
0.7	0.020 (0.014)	0.037 (0.008)	**0.010 (0.007) **	0.024 (27.8%)	0.038 (0.6%)	**0.013 (71.6%)**
1	0.021 (0.015)	0.008 (0.006)	**0.008 (0.006)**	0.026 (19.6%)	0.010 (37.2%)	**0.010 (43.2%)**
1.3	0.020 (0.015)	0.028 (0.011)	**0.007 (0.006)**	0.025 (20.4%)	0.030 (6.8%)	**0.009 (72.8%)**
1.5	0.020 (0.015)	0.049 (0.012)	**0.007 (0.005)**	0.025 (23.8%)	0.050 (0.2%)	**0.009 (76.0%)**
**ER network,** P(A)=0.1						
0.7	0.025 (0.018)	0.027 (0.008)	**0.009 (0.007)**	0.030 (18.2%)	0.028 (6.8%)	**0.011 (75.0%)**
1	0.022 (0.016)	0.008 (0.006)	**0.007 (0.006)**	0.027 (14.2%)	0.010 (37.4%)	**0.009 (48.4%)**
1.3	0.022 (0.017)	0.026 (0.011)	**0.007 (0.006)**	0.028 (17.6%)	0.028 (6.8%)	**0.009 (75.6%)**
1.5	0.022 (0.017)	0.043 (0.012)	**0.007 (0.005)**	0.027 (19.6%)	0.045 (0.8%)	**0.009 (79.6%)**

**Note:** Bold values indicate the best performance (lowest absolute Bias or RMSE) in each row.

**Table 3:** Bias (SD) and RMSE (P^best^) for different sampling strategies in synthetic networks.

Sampling Strategy	Bias (SD)			RMSE (P^best^)		
	**Sample**	**ECM**	** ${\mathrm{ECM}}_{\mathrm{ac}}$ **	**Sample**	**ECM**	** ${\mathrm{ECM}}_{\mathrm{ac}}$ **
**BA network,** P(A)=0.1						
F	0.021 (0.016)	0.092 (0.014)	**0.011 (0.008)**	0.026 (29.1%)	0.094 (5.8%)	**0.013 (65.1%)**
P5	0.018 (0.014)	0.092 (0.013)	**0.015 (0.010)**	0.022 (41.8%)	0.093 (5.8%)	**0.018 (52.4%)**
P10	0.019 (0.015)	0.092 (0.013)	**0.011 (0.009)**	0.024 (33.0%)	0.094 (5.4%)	**0.014 (61.6%)**
W	0.021 (0.016)	0.092 (0.014)	**0.010 (0.008)**	0.026 (28.1%)	0.094 (5.7%)	**0.013 (66.1%)**
**ER network,** P(A)=0.1						
F	0.032 (0.024)	0.059 (0.012)	**0.010 (0.008)**	0.040 (19.4%)	0.061 (8.1%)	**0.013 (72.6%)**
P5	0.023 (0.017)	0.059 (0.012)	**0.010 (0.008)**	0.029 (26.9%)	0.060 (7.8%)	**0.013 (65.3%)**
P10	0.030 (0.022)	0.059 (0.012)	**0.010 (0.008)**	0.037 (20.6%)	0.060 (8.1%)	**0.013 (71.2%)**
W	0.032 (0.024)	0.059 (0.012)	**0.010 (0.008)**	0.040 (20.0%)	0.061 (7.7%)	**0.013 (72.3%)**

**Note:** Bold values indicate the best performance (lowest absolute Bias or RMSE) in each row.

**Table 4:** ${\mathrm{ECM}}_{\mathrm{ac}}$ coverage rates at the 90% nominal level. Each cell shows the coverage rates for BS-Ego (BS-Tree, BS-Pool). The value closest to the nominal 0.90 level in each cell is bolded.

PA	AR = 0.8	AR = 1.0	AR = 1.2	AR = 1.4	AR = 1.6	AR = 1.8
0.10	**0.86** (0.95, 0.85)	0.83 (**0.94**, 0.83)	0.85 (0.98, **0.86**)	0.93 (0.99, **0.91**)	**0.92** (0.99, 0.92)	**0.88** (0.97, 0.87)
0.20	0.75 (**0.92**, 0.75)	0.83 (**0.95**, 0.77)	**0.90** (0.96, 0.89)	0.83 (0.97, **0.88**)	0.78 (**0.94**, 0.79)	0.81 (**0.90**, 0.81)
0.30	0.84 (**0.95**, 0.77)	0.84 (0.98, **0.86**)	**0.90** (0.97, 0.91)	**0.85** (0.98, 0.84)	0.83 (**0.95**, 0.84)	**0.89** (0.98, 0.88)
0.40	0.80 (**0.93**, 0.84)	0.86 (0.98, **0.88**)	**0.85** (0.96, 0.84)	0.84 (**0.94**, 0.84)	0.81 (**0.94**, 0.84)	0.82 (**0.94**, 0.83)

**Note:** Bold values indicate the best performance (lowest absolute Bias or RMSE) in each row.

**Table 5:** ${\mathrm{ECM}}_{\mathrm{ac}}$ coverage rates at the 95% nominal level. Each cell shows the coverage rates for BS-Ego (BS-Tree, BS-Pool). The value closest to the nominal 0.95 level in each cell is bolded.

PA	AR = 0.8	AR = 1.0	AR = 1.2	AR = 1.4	AR = 1.6	AR = 1.8
0.10	0.91 (**0.98**, 0.91)	0.89 (**0.98**, 0.90)	**0.96** (1.00, 0.96)	**0.96** (1.00, 0.97)	**0.93** (0.99, 0.92)	**0.95** (0.99, 0.95)
0.20	0.90 (0.99, **0.91**)	0.91 (0.98, **0.92**)	**0.97** (0.99, 0.97)	0.97 (0.99, **0.96**)	**0.93** (0.99, 0.93)	0.91 (0.99, **0.93**)
0.30	0.87 (**0.98**, 0.86)	0.97 (1.00, **0.96**)	**0.95** (0.99, 0.95)	0.96 (1.00, **0.95**)	**0.95** (0.99, 0.96)	0.89 (**0.99**, 0.90)
0.40	**0.93** (0.99, 0.92)	**0.93** (0.98, 0.92)	**0.93** (0.99, 0.91)	**0.94** (0.99, 0.93)	**0.92** (0.99, 0.92)	0.85 (**0.97**, 0.91)

**Note:** Bold values indicate the best performance (lowest absolute Bias or RMSE) in each row.

**Figure A1:**
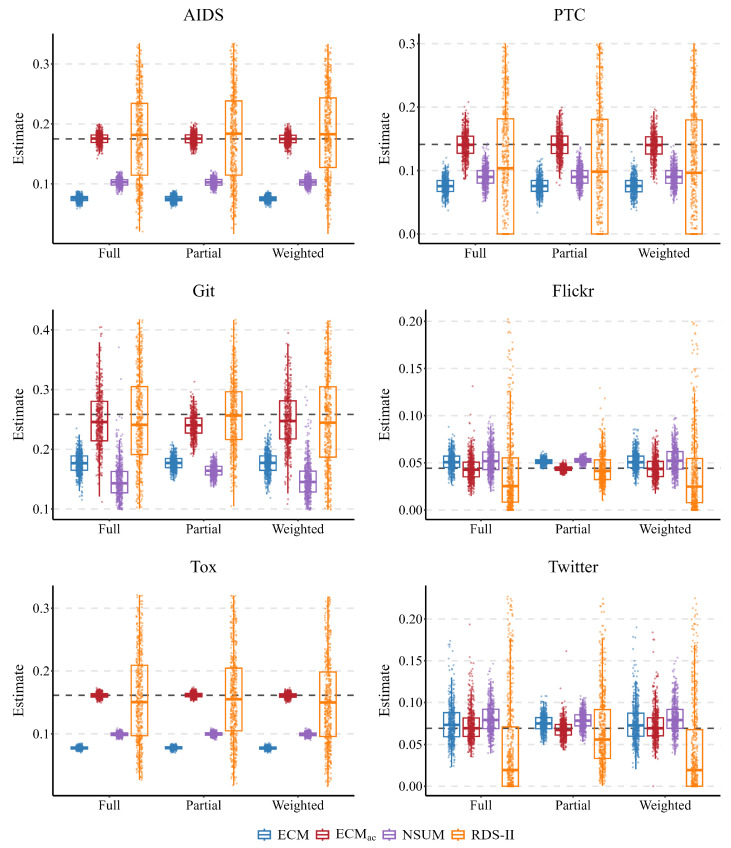
Comparison of ECM, ${\mathrm{ECM}}_{\mathrm{ac}}$, NSUM, and RDS-II on several real-world networks under different sampling strategies (Full, Partial, and Weighted). Each panel corresponds to a distinct network. The dashed horizontal line indicates the true population proportion. ${\mathrm{ECM}}_{\mathrm{ac}}$ exhibits tighter concentration around the true value and reduced bias compared with the baselines across most networks and sampling regimes.

**Figure A2:**
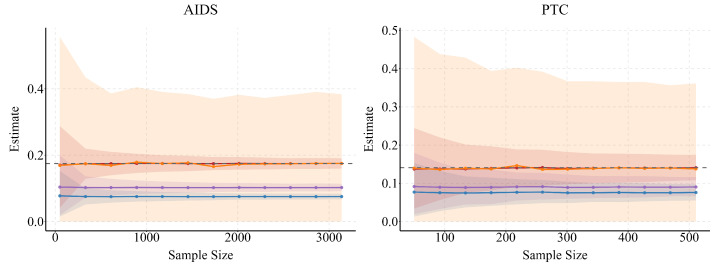

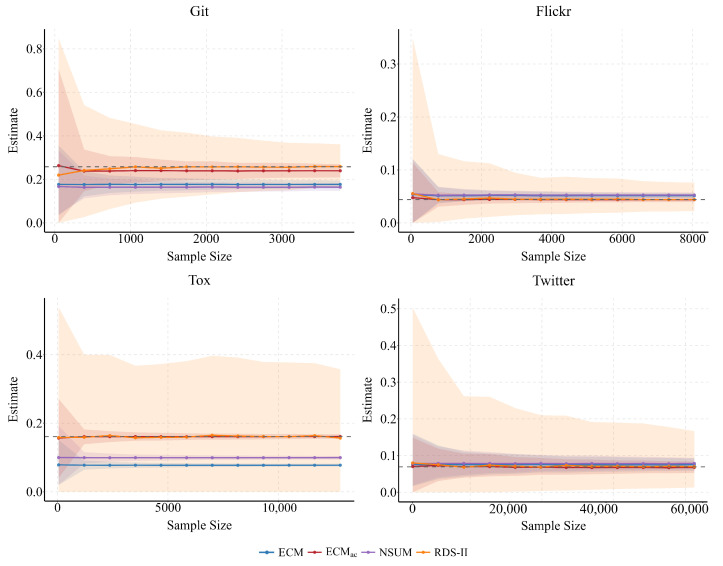
Performance of ECM, ${\mathrm{ECM}}_{\mathrm{ac}}$, NSUM, and RDS-II as a function of sample size on representative real-world networks. The dashed horizontal line indicates the true population proportion. Solid lines denote mean estimates across repetitions, while shaded regions indicate empirical variability. ${\mathrm{ECM}}_{\mathrm{ac}}$ shows faster convergence and improved stability relative to the baselines as sample size increases.

## Data Availability

All data and materials used in this study are publicly available. The datasets can be accessed from their original repositories as cited in the main text. The full code required to reproduce all simulations and figures is available on GitHub at https://github.com/kkangyoudianhan/Peer-Reporting-Sampling-Design-and-Unbiased-Estimates (accessed on 11 November 2025. All analyses were conducted using Python 3.8).

## Supplementary Materials

The following supporting information can be downloaded at https://www.mdpi.com/article/10.3390/e28010116/s1, Table S1: Supporting results for Section 5.1 (Population Proportion P(A)). Representative ECM and ECM_*ac*_ estimates, biases, and network parameters for BA(AR-0.7); Table S2: Supporting results for Section 5.2 (Activity Ratio (AR)). Mean, SD, and 90% CI of ECM and ECM_*ac*_ across activity ratios; Table S3: Supporting results for Section 5.5 (Combined Effects of AR and P(A)). Mean bias of ECM and ECM_*ac*_ under P5 sampling; Table S4: Estimator error and 90% central range across homophily (H) and activity ratio (AR) settings—BA network; Table S5: Summary statistics for ECM and ECM_*ac*_ on the six networks, broken down by sampling strategy. References [41,42] are cited in the Supplementary Materials.

## Appendix A. Variance Estimation

### Appendix A.1. Asymptotic Variance Estimation

An analytical approximation for the variance of the adjusted estimator can be derived using a first-order Taylor expansion, commonly known as the Delta method [43]. This approach provides theoretical insight into the estimator’s variability.

Let the function be $g\left(\hat{p}\left(x\right),\hat{AR}\right)={\hat{P}}_{\mathrm{ac}}\left(A\right)$, where $\hat{p}\left(x\right)={\hat{P}}_{\mathrm{ECM}}\left(A\right)$. The asymptotic variance of this function can be approximated as

(A1) $$\mathrm{Var}\;\left({\hat{P}}_{\mathrm{ac}}\left(A\right)\right)\;\approx \;{\left(\frac{\partial g}{\partial p(x)}\right)}^{2}\mathrm{Var}\left(\hat{p}\left(x\right)\right)+{\left(\frac{\partial g}{\partial AR}\right)}^{2}\mathrm{Var}\left(\hat{AR}\right)+2\;\left(\frac{\partial g}{\partial p(x)}\right)\left(\frac{\partial g}{\partial AR}\right)\mathrm{Cov}\left(\hat{p}\left(x\right),\hat{AR}\right),$$

where the partial derivatives are evaluated at the true population values (p(x),AR).

In practice, using this formula requires sample-based estimates for the variance and covariance terms. Deriving closed-form expressions for these terms is non-trivial due to the complex dependence structure of ego-network samples. Therefore, due to these practical challenges, we primarily rely on the more direct and robust nonparametric bootstrap methods detailed in Appendix A.2 for constructing confidence intervals in our experiments.

### Appendix A.2. Bootstrap Methods for Confidence

This appendix provides technical details for the three bootstrap resampling schemes evaluated in Section 4.3. Let the original sample consist of a set of *S* egos, S={1,…,S}. For each ego i∈S, we observe its attribute $Z_{i}$, degree $k_{i}$, and the list of reported neighbors $N_{i}$.

#### Appendix A.2.1. Resampling Designs

The three designs progressively relax the independence assumptions in ego-centric data—from the simplest (BS-Ego) to the most granular (BS-Pool).

(a) Ego-level Resampling (BS-Ego). This is the standard nonparametric bootstrap that treats each ego-network as a single independent sampling unit.
  i. Draw a bootstrap sample of egos $S^{*}$ by sampling *S* egos from S with replacement.
  ii. For each $i^{*}\in S^{*}$, include its complete observed record (degree and alter list).
  iii. Recompute the estimator ${\hat{P}}_{\mathrm{ac}}^{*}\left(A\right)$ using Equation (17) on the resampled ego set.
  This approach preserves within-ego dependence but ignores the uncertainty arising from the second-stage alter sampling. It serves as the default bootstrap when no neighbor-level information is available.
(b) Hierarchical Tree Bootstrap (BS-Tree). A two-level resampling scheme that mirrors the hierarchical structure of ego-centric sampling. Each ego is kept fixed, while its alter list is resampled to capture within-ego stochasticity.
  i. Level 1 (Ego layer): Retain all *S* egos from the original sample.
  ii. Level 2 (Alter layer): For each ego i∈S, resample alters with replacement from its own alter list to form a bootstrap neighborhood $N_{i}^{*}$.
  iii. Recompute ${\hat{P}}_{\mathrm{ac}}^{*}\left(A\right)$ on the reconstructed bootstrap dataset.
(c) Neighbor Pool Bootstrap (BS-Pool). This design resamples at the *edge level*, ignoring ego boundaries. To maintain directionality, edges are divided into two origin-based groups: those emanating from *A*-egos and those from *B*-egos. Within each group, alters are treated as conditionally i.i.d.
  i. Construct two disjoint edge pools:
    $$E_{A}=\left\{\mathrm{edges}\;\mathrm{from}\;\mathrm{sampled}\;A-\mathrm{egos}\right\},\;E_{B}=\left\{\mathrm{edges}\;\mathrm{from}\;\mathrm{sampled}\;B-\mathrm{egos}\right\}.$$
    Code each edge in $E_{A}$ as $Y_{A\to B}\in \left\{0,1\right\}$ (1 if the alter is *B*), and each edge in $E_{B}$ as $Y_{B\to A}\in \left\{0,1\right\}$ (1 if the alter is *A*). Let $s_{A}=\left|E_{A}\right|$, $s_{B}=\left|E_{B}\right|$, and $m_{AB}=\sum_{E_{A}}Y_{A\to B}$, $m_{BA}=\sum_{E_{B}}Y_{B\to A}$.
  ii. For each bootstrap replication, draw $s_{A}$ samples with replacement from $E_{A}$ and $s_{B}$ from $E_{B}$, obtaining
    $$m_{AB}^{*}=\sum Y_{A\to B}^{*},\;m_{BA}^{*}=\sum Y_{B\to A}^{*}.$$
    Each draw corresponds to one resampled edge indicator from the respective pool.
  iii. Compute the resampled proportions ${\hat{P}}_{AB}^{*(S)}=m_{AB}^{*}/s_{A},\;{\hat{P}}_{BA}^{*(S)}=m_{BA}^{*}/s_{B},$ and, keeping the sample activity ratio fixed, recompute ${\hat{P}}_{\mathrm{ac}}^{*}\left(A\right)$.
  This edge-level approach does not account for correlations within each ego’s neighborhood, but it avoids mixing edges that originate from different groups, which could otherwise distort the estimates of $P_{AB}$ and $P_{BA}$. Bootstrap samples in which no edges are drawn from either *A*- or *B*-egos ($s_{A}=0$ or $s_{B}=0$) are excluded from analysis. To prevent numerical errors when proportions are extremely close to 0 or 1, the resampled proportions are bounded within ${\hat{P}}_{AB}^{*(S)},{\hat{P}}_{BA}^{*(S)}\in \left[10^{-6},\;1-10^{-6}\right]$, while the reported point estimates use the original, unbounded values.

#### Appendix A.2.2. Confidence Interval Construction

For each bootstrap design, the overall procedure is as follows:

1. Compute the point estimate ${\hat{P}}_{\mathrm{ac}}\left(A\right)$ from the original sample.
2. For b=1,2,…,B:
  (i) Generate a bootstrap sample using one of the three designs: BS-Ego, BS-Tree, or BS-Pool.
  (ii) Recompute the estimator on this replicate, denoted ${\hat{P}}_{\mathrm{ac}}^{*(b)}\left(A\right)$.
3. Sort the bootstrap estimates in ascending order:
  $${\hat{P}}_{\mathrm{ac}}^{*(1)}\left(A\right)\leq {\hat{P}}_{\mathrm{ac}}^{*(2)}\left(A\right)\leq \ldots \leq {\hat{P}}_{\mathrm{ac}}^{*(B)}\left(A\right).$$
4. Construct the (1−α) percentile confidence interval as
  $${\mathrm{CI}}_{1-\alpha }=\left[{\hat{P}}_{\mathrm{ac}}^{*(\lceil \alpha B/2\rceil )}\left(A\right),{\hat{P}}_{\mathrm{ac}}^{*(\lceil (1-\alpha /2)B\rceil )}\left(A\right)\right].$$

## Appendix B. Supplementary Comparison with RDS and NSUM

This appendix reports supplementary comparisons between the proposed ${\mathrm{ECM}}_{\mathrm{ac}}$ estimator and two representative baseline approaches, RDS-II and NSUM, on the same set of real-world networks. We emphasize that these baselines are designed for different sampling mechanisms and inferential targets (e.g., respondent-driven settings or scale-up style inference), and therefore the results below should be interpreted as qualitative reference rather than as a definitive ranking.

Figure A1 compares the empirical distributions of estimates under three ego-centric reporting regimes (Full, Partial, and Weighted) across multiple networks. Across panels, ${\mathrm{ECM}}_{\mathrm{ac}}$ is generally more concentrated around the true proportion (dashed line), indicating improved stability and reduced bias relative to the conventional ECM. In contrast, ECM exhibits systematic deviations in networks where degree–attribute imbalance is present, while NSUM and RDS-II show noticeably larger dispersion in several cases, reflecting their mismatch with the ego-centric sampling context.

Figure A2 further examines how estimates evolve with increasing sample size. The curves suggest that ${\mathrm{ECM}}_{\mathrm{ac}}$ converges faster toward the ground-truth proportion and maintains narrower empirical variability bands. By comparison, the baseline methods tend to converge more slowly and stabilize at values that remain offset from the benchmark in some networks, consistent with the fact that their estimands and inclusion mechanisms differ from the ego-centric design studied in this paper.
