# Supplementary material for: Peer Reporting: Sampling Design and Unbiased Estimates
Source: Entropy (Basel). 2026 Jan 18;28(1):116. doi: 10.3390/e28010116 (PMC12840051; doi:10.3390/e28010116)
Supplement: Supplementary file 1 [file entropy-28-00116-s001.zip › entropy-4052984-supplementary.pdf]

Supporting Information for
Peer Reporting: Sampling Design and Unbiased Estimates

Kang Wen, Jianhong Mou and Xin Lu *

College of Systems Engineering, National University of Defense Technology, Changsha 410073, China; wenkang@nudt.edu.cn (K.W.); moujianhong@nudt.edu.cn (J.M.)
***** Correspondence: lvxin@nudt.edu.cn

This PDF file includes:

Tables S1 to S5

Supporting Information Text

Tables

Table S1. Supporting results for Section 5.1 (Population Proportion P(A)). Representative ECM and ECM$_{ac}$ estimates, biases, and network parameters for BA(AR-0.7).

Network setting	$P(A)$	R	ECM	ECM$_{ac}$	ECM bias	ECM$_{ac}$bias
BA, $AR = 0.7$, $P(A) = 0.10$	0.10	0.749	0.070	0.091	-0.030	-0.009
BA, $AR = 0.7$, $P(A) = 0.20$	0.20	0.749	0.145	0.185	-0.055	-0.015
BA, $AR = 0.7$, $P(A) = 0.30$	0.30	0.749	0.225	0.279	-0.075	-0.021
BA, $AR = 0.7$, $P(A) = 0.40$	0.40	0.750	0.315	0.380	-0.085	-0.020

Table S2. Supporting results for Section 5.2 (Activity Ratio (AR)). Mean, SD, and 90% CI of ECM and ECM$_{ac}$ across activity ratios.

AR	ECM mean	ECM SD	ECM$_{ac}$ mean	ECM$_{ac}$ SD	ECM 90% CI	ECM$_{ac}$ 90% CI
0.5	0.1702	0.0115	0.0722	0.0179	[0.1696, 0.1708]	[0.0712, 0.0731]
0.6	0.1290	0.0132	0.0460	0.0174	[0.1284, 0.1297]	[0.0451, 0.0469]
0.7	0.0903	0.0147	0.0278	0.0156	[0.0895, 0.0911]	[0.0270, 0.0287]
0.8	0.0564	0.0159	0.0185	0.0129	[0.0556, 0.0573]	[0.0179, 0.0192]
0.9	0.0257	0.0149	0.0142	0.0105	[0.0249, 0.0265]	[0.0137, 0.0148]
1.0	0.0131	0.0098	0.0131	0.0098	[0.0126, 0.0136]	[0.0126, 0.0136]
1.1	0.0226	0.0149	0.0131	0.0099	[0.0218, 0.0234]	[0.0126, 0.0136]
1.2	0.0447	0.0176	0.0139	0.0109	[0.0438, 0.0456]	[0.0133, 0.0144]
1.3	0.0687	0.0178	0.0159	0.0114	[0.0678, 0.0696]	[0.0153, 0.0165]
1.4	0.0918	0.0182	0.0186	0.0129	[0.0909, 0.0928]	[0.0180, 0.0193]
1.5	0.1079	0.0179	0.0182	0.0127	[0.1070, 0.1088]	[0.0175, 0.0188]

Table S3. Supporting results for Section 5.5 (Combined Effects of AR and P(A)). Mean bias of ECM and ECM$_{ac}$ under P5 sampling.

AR	P(A)=0.10		P(A)=0.15		P(A)=0.20		P(A)=0.25	
	ECM	ECM$_{ac}$	ECM	ECM$_{ac}$	ECM	ECM$_{ac}$	ECM	ECM$_{ac}$
0.5	0.0609	0.0236	0.0879	0.0280	0.1093	0.0280	0.1285	0.0244
0.7	0.0382	0.0152	0.0503	0.0159	0.0619	0.0173	0.0739	0.0194
1.0	0.0086	0.0089	0.0089	0.0093	0.0103	0.0103	0.0119	0.0121
1.5	0.0494	0.0080	0.0701	0.0110	0.0867	0.0122	0.1030	0.0152
2.0	0.0938	0.0087	0.1310	0.0110	0.1595	0.0132	0.1806	0.0149
2.5	0.1386	0.0095	0.1852	0.0112	0.2180	0.0124	0.2421	0.0130
AR	P(A)=0.30		P(A)=0.35		P(A)=0.40		P(A)=0.45	
	ECM	ECM$_{ac}$	ECM	ECM$_{ac}$	ECM	ECM$_{ac}$	ECM	ECM$_{ac}$
0.5	0.1486	0.0264	0.1614	0.0242	0.1732	0.0265	0.1855	0.0290
0.7	0.0801	0.0189	0.0874	0.0189	0.0911	0.0183	0.0953	0.0160
1.0	0.0118	0.0116	0.0132	0.0124	0.0149	0.0135	0.0139	0.0123
1.5	0.1143	0.0165	0.1235	0.0171	0.1277	0.0173	0.1280	0.0166
2.0	0.1965	0.0152	0.2064	0.0156	0.2127	0.0175	0.2127	0.0188
2.5	0.2599	0.0146	0.2678	0.0141	0.2692	0.0150	0.2642	0.0155

Table S4. Estimator error and 90% central range across homophily (H) and activity ratio (AR) settings — BA network

H	AR	Error (P10)		Error (P5)		90% central range	
		ECM	ECM_{ac}	ECM	ECM_{ac}	ECM	ECM_{ac}
-0.30	0.5	-0.1774	-0.0578	-0.1917	-0.0765	[-0.1917, -0.1774]	[-0.0765, -0.0578]
-0.30	0.7	-0.0844	-0.0192	-0.0987	-0.0348	[-0.0987, -0.0844]	[-0.0348, -0.0192]
-0.30	1.3	0.0528	-0.0022	0.0334	-0.0209	[0.0334, 0.0528]	[-0.0209, -0.0022]
-0.30	1.5	0.1028	0.0108	0.0817	-0.0096	[0.0817, 0.1028]	[-0.0096, 0.0108]
-0.30	2.5	0.2768	0.0610	0.2576	0.0396	[0.2576, 0.2768]	[0.0396, 0.0610]
-0.20	0.5	-0.1730	-0.0521	-0.1877	-0.0712	[-0.1877, -0.1730]	[-0.0712, -0.0521]
-0.20	0.7	-0.0841	-0.0190	-0.0993	-0.0357	[-0.0993, -0.0841]	[-0.0357, -0.0190]
-0.20	1.3	0.0552	0.0006	0.0348	-0.0191	[0.0348, 0.0552]	[-0.0191, 0.0006]
-0.20	1.5	0.0993	0.0075	0.0783	-0.0127	[0.0783, 0.0993]	[-0.0127, 0.0075]
-0.20	2.5	0.2655	0.0483	0.2426	0.0235	[0.2426, 0.2655]	[0.0235, 0.0483]
-0.10	0.5	-0.1741	-0.0535	-0.1880	-0.0716	[-0.1880, -0.1741]	[-0.0716, -0.0535]
-0.10	0.7	-0.0877	-0.0228	-0.1031	-0.0398	[-0.1031, -0.0877]	[-0.0398, -0.0228]
-0.10	1.3	0.0566	0.0017	0.0376	-0.0166	[0.0376, 0.0566]	[-0.0166, 0.0017]
-0.10	1.5	0.1002	0.0083	0.0786	-0.0125	[0.0786, 0.1002]	[-0.0125, 0.0083]
-0.10	2.5	0.2594	0.0416	0.2359	0.0164	[0.2359, 0.2594]	[0.0164, 0.0416]
0.00	0.5	-0.1675	-0.0449	-0.1796	-0.0606	[-0.1796, -0.1675]	[-0.0606, -0.0449]
0.00	0.7	-0.0880	-0.0232	-0.1032	-0.0398	[-0.1032, -0.0880]	[-0.0398, -0.0232]
0.00	1.3	0.0589	0.0038	0.0405	-0.0140	[0.0405, 0.0589]	[-0.0140, 0.0038]
0.00	1.5	0.1024	0.0104	0.0826	-0.0087	[0.0826, 0.1024]	[-0.0087, 0.0104]
0.00	2.5	0.2396	0.0202	0.2145	-0.0056	[0.2145, 0.2396]	[-0.0056, 0.0202]
0.20	0.5	-0.1272	0.0055	-0.1386	-0.0085	[-0.1386, -0.1272]	[-0.0085, 0.0055]
0.20	0.7	-0.0738	0.0019	-0.0881	-0.0233	[-0.0881, -0.0738]	[-0.0233, 0.0019]
0.20	1.3	0.0496	-0.0050	0.0304	-0.0235	[0.0304, 0.0496]	[-0.0235, -0.0050]
0.20	1.5	0.0838	-0.0073	0.0636	-0.0265	[0.0636, 0.0838]	[-0.0265, -0.0073]
0.20	2.5	0.1821	-0.0374	0.1540	-0.0634	[0.1540, 0.1821]	[-0.0634, -0.0374]

Table S5. Summary statistics for ECM and ECM_{ac} on the six networks, broken down by sampling strategy.

Networks	Strategy	Estimator	Mean	SD	90% CI	Networks	Mean	SD	90% CI
AIDS	F	ECM	0.075	0.014	[0.059, 0.092]	PTC	0.076	0.023	[0.042, 0.114]
		ECM_{ac}	0.174	0.025	[0.143, 0.205]		0.140	0.034	[0.086, 0.194]
	P10	ECM	0.075	0.014	[0.058, 0.092]		0.076	0.023	[0.042, 0.114]
		ECM_{ac}	0.174	0.025	[0.141, 0.206]		0.140	0.034	[0.087, 0.194]
	P5	ECM	0.076	0.014	[0.058, 0.093]		0.076	0.022	[0.042, 0.113]
		ECM_{ac}	0.174	0.026	[0.139, 0.206]		0.140	0.034	[0.087, 0.194]

Networks	Strategy	Estimator	Mean	SD	90% CI	Networks	Mean	SD	90% CI
Git	W	ECM	0.075	0.014	[0.058, 0.092]	Flickr	0.076	0.023	[0.042, 0.113]
		ECM_{ac}	0.174	0.025	[0.142, 0.204]		0.139	0.034	[0.086, 0.192]
	F	ECM	0.176	0.047	[0.112, 0.238]		0.052	0.021	[0.023, 0.082]
		ECM_{ac}	0.254	0.098	[0.138, 0.395]		0.044	0.017	[0.022, 0.073]
	P10	ECM	0.177	0.033	[0.136, 0.217]		0.051	0.011	[0.040, 0.062]
		ECM_{ac}	0.242	0.067	[0.175, 0.302]		0.044	0.007	[0.037, 0.052]
	P5	ECM	0.177	0.028	[0.143, 0.211]		0.051	0.011	[0.040, 0.061]
		ECM_{ac}	0.237	0.056	[0.181, 0.292]		0.043	0.007	[0.036, 0.051]
	W	ECM	0.176	0.048	[0.107, 0.237]		0.052	0.021	[0.024, 0.082]
		ECM_{ac}	0.253	0.095	[0.131, 0.389]		0.045	0.017	[0.021, 0.074]
Tox	W	ECM	0.077	0.012	[0.067, 0.087]	Twitter	0.063	0.013	[0.057, 0.068]
		ECM_{ac}	0.160	0.019	[0.142, 0.175]		0.033	0.004	[0.032, 0.035]
	F	ECM	0.078	0.013	[0.068, 0.087]		0.063	0.013	[0.056, 0.068]
		ECM_{ac}	0.160	0.021	[0.145, 0.176]		0.033	0.004	[0.031, 0.035]
	P10	ECM	0.078	0.012	[0.068, 0.087]		0.063	0.012	[0.058, 0.068]
		ECM_{ac}	0.161	0.020	[0.144, 0.177]		0.034	0.004	[0.032, 0.035]
	P5	ECM	0.077	0.012	[0.067, 0.087]		0.063	0.013	[0.057, 0.068]
		ECM_{ac}	0.160	0.020	[0.142, 0.176]		0.033	0.004	[0.031, 0.035]

References

1. Spiller M W, Gile K J, Handcock M S, et al. Evaluating variance estimators for respondent-driven sampling[J]. Journal of survey statistics and methodology, 2018, 6(1): 23-45.
2. Lu X, Malmros J, Liljeros F, et al. Respondent-driven sampling on directed networks[J]. 2013.
3. Gile K J, Beaudry I S, Handcock M S, et al. Methods for inference from respondent-driven sampling data[J]. Annual Review of Statistics and Its Application, 2018, 5(1): 65-93.
4. Baraff A J, McCormick T H, Raftery A E. Estimating uncertainty in respondent-driven sampling using a tree bootstrap method[J]. Proceedings of the National Academy of Sciences, 2016, 113(51): 14668-14673.
